# Meeting Prevention Beyond Awareness: A Qualitative Study Exploring Attitudes and Beliefs Towards Dating Violence and Prevention Among Emerging Adults

**DOI:** 10.3390/ijerph23030294

**Published:** 2026-02-27

**Authors:** Ana Cristina Saial, Liliana Faria, Alda Portugal, Élvio Rubio Gouveia, Miguel Campos, Ana Paula Relvas

**Affiliations:** 1 Laboratory for Robotics and Engineering Systems (LARSyS), Interactive Technologies Institute (ITI), 1649-003 Lisbon, Portugal; erubiog@staff.uma.pt; 2 Faculty of Psychology and Educational Sciences, University of Coimbra, 3000-115 Coimbra, Portugal; uc2020230750@student.uc.pt (L.F.); aprelvas@fpce.uc.pt (A.P.R.); 3 Centre for Social Studies, University of Coimbra, 3000-995 Coimbra, Portugal; alda.portugal@staff.uma.pt; 4 Faculty of Psychology, University of Lisbon, 1649-013 Lisbon, Portugal; 5 Faculty of Social Sciences, Department of Physical Education and Sports, University of Madeira, 9020-105 Funchal, Portugal; 6 WowSystems Informática Lda., 9050-446 Funchal, Portugal; miguel.campos@wowsystems.pt

**Keywords:** dating violence, emerging adulthood, attitudes and beliefs, romantic love myths, preventive interventions

## Abstract

**Highlights:**

**Public health relevance—How does this work relate to a public health issue?**

- Dating violence among emerging adults is a significant public health concern, with high prevalence rates (around 80% of Portuguese university students) and serious consequences for physical and mental health, including anxiety, depression, and risky sexual behavior, among others.
- This study addresses a critical gap and reveals an important paradox: despite high awareness and knowledge about the various types of dating violence and their severity, participants exhibit an attitude–behavior inconsistency that perpetuates the normalization of abusive behaviors.

**Public health significance—Why is this work of significance to public health?**

- By showing that emerging adults experience cognitive dissonance between their general knowledge about dating violence and their own personal experiences, this study reveals that awareness and knowledge alone are insufficient to prevent dating violence. Some of the barriers identified were myths of romantic love and difficulty recognizing psychological violence behaviors.
- The findings of this study highlight that traditional knowledge-focused approaches should be replaced with behavioral-change interventions that address emotional regulation, interpersonal skills, gender norms, and social factors that normalize dating violence.

**Public health implications—What are the key implications or messages for practitioners, policy makers and/or researchers in public health?**

- Practitioners and policy makers should redesign school-based prevention interventions and community programs, adapting them to the developmental stage and context of emerging adults. Prevention interventions should focus on developing healthy interpersonal relationship skills, such as social and emotional skills, be regular, and be integrated into formal education curricula.
- Researchers and practitioners should prioritize co-design flexible, accessible, and scalable prevention approaches, such as technological tools (including mobile applications, games, and digital platforms) that align with the emerging adults’ interests and lived realities, as evidence suggests that digital tools can effectively promote engagement, empathy, self-reflection and reduce social norms that support dating violence.

**Abstract:**

Dating violence (DV) is an increasingly prevalent phenomenon among emerging adults (aged 18–25 years), and the relationship between awareness and behavior remains poorly understood. This study explores emerging adults’ attitudes and beliefs toward DV and summarizes recommendations for designing prevention programs. A qualitative study using three focus groups (n = 16 emerging adults aged 18–25; 56% female) was conducted. Data were collected via semi-structured interviews and analyzed using thematic analysis. Three main themes emerged: (1) gender roles, (2) healthy intimate relationships, and (3) dating violence. Participants demonstrated high awareness of DV types, severity, and prevalence. However, they also exhibited an attitude–behavior inconsistency, reflected in the normalization and excusing of violence, and difficulty recognizing violent situations in their own relationships. Myths of romantic love and cognitive dissonance between general knowledge and personal experience create barriers to recognizing abuse—particularly psychological abuse, which is often confused with concern. Participants suggested integrating prevention strategies into schools and communities, with interventions tailored to their interests and realities (e.g., mobile applications, games and social media awareness campaigns). This study reveals that awareness and knowledge alone are insufficient for prevention. Efforts should shift from a knowledge-focused to a behavior-change approach, promoting emotional regulation, interpersonal skills, and addressing social and gender norms. Relevant implications for practice and preventive intervention design are discussed.

## 1. Introduction

Dating violence (DV), a type of intimate partner violence (IPV) that occurs in romantic relationships during courtship, constitutes a significant public health concern affecting millions of young people worldwide [1,2]. The World Health Organization (WHO) defines DV as behavior by an intimate partner or ex-partner that causes physical, sexual, or psychological harm, including physical aggression, sexual coercion, psychological abuse, and controlling behaviors [3,4]. In emerging adulthood, a phase encompassing individuals aged 18–25 years and characterized by identity formation and the exploration and experimentation of intimate and romantic relationships [5], there has been a notable increase in the prevalence of DV [6,7,8,9]. In Portugal, specifically, a study of university students showed that around 80% of the sample admitted to having been a victim or perpetrator of abusive behavior [6]. Similarly, the study by Saial et al. [7] conducted with emerging adults indicated that 62.9% of the sample reported having suffered cyberdating abuse and 74.3% perpetrated it, with an emphasis on controlling behaviors.

The importance of DV in public health goes beyond its prevalence; it represents a serious problem for the physical and mental health of those involved [2,9]. DV is associated with high risks of depression, anxiety, post-traumatic stress disorder, sleep disorders, risky sexual behavior, sexually transmitted diseases, unwanted pregnancies, and sexual health problems [3,10,11,12]. At a societal level, DV perpetuates cycles of violence, predicts violent behavior in adult romantic relationships, normalizes dysfunctional behavior, and establishes patterns of inappropriate behavior that prevail into adulthood [13,14,15,16]. Furthermore, despite the impacts of the high prevalence of psychological violence in dating relationships (percentages reaching 95%), it continues to be minimized and normalized by both victims and perpetrators [17,18,19]. DV is a human rights violation that is punishable by law and requires effective preventive interventions [20].

### 1.1. Emerging Adulthood and Romantic Relationships

Emerging adulthood is a distinct developmental period within industrialized societies, characterized by significant transitions in education, employment, residence, and intimate relationships [5,21]. During this phase, young people typically leave parental households, develop a sense of identity and autonomy, and engage extensively in romantic relationships [5,21]. The ability to form and maintain a romantic relationship is one of the most important tasks of emerging adulthood [22]. Romantic relationships among emerging adults have changed considerably over the past few decades, reflecting cultural shifts that value individualism, personal fulfilment, autonomy, and freedom [23]. Unlike previous generations, for whom marriage and parenthood were expected by the mid-twenties, currently emerging adults prioritize education and career development [24,25]. In Portugal, the average age of women and men at first marriage in 2024 is 34.3 and 35.8 years, respectively [26], and the average age of women at the birth of their first child is 30.3 years [27]. Compared to 1995, Portuguese people are postponing marriage by about 10 years, and the birth of their first child occurs five years later [26,27]. Another major change in Portugal is the transition to entering the labor market, as young people face great difficulties in finding their first job, leading to longer periods of inactivity [25,28] and remaining in their parents’ home for longer due to economic difficulties [29].

All of this reflects the diversity of experiences that currently exist in emerging adulthood. All the instability associated with this phase, exacerbated by social gender norms, sets the stage for situations of vulnerability, such as violence in relationships.

### 1.2. Dating Violence: Types, Prevalence, and Risk Factors

The Centers for Disease Control and Prevention (CDC) identifies four types of intimate partner violence: (1) physical violence, attempting to cause injury using physical force; (2) sexual violence, forcing or attempting to force participation in sexual acts without consent; (3) stalking, unwanted and persistent contact causing fear or concern for the safety of the victim or someone close to them; and (4) psychological aggression, use of verbal and non-verbal communication intended to cause mental/emotional harm or control a partner [30]. Among these, psychological abuse emerges as the most significant form, followed by physical violence and sexual coercion [9]. Several studies indicate that more than half of emerging adults have used some form of violence in their romantic relationships [6,7,9,31].

Research indicates that DV is a bidirectional phenomenon, with similar proportions of men and women engaging in at least some form of violence [32,33,34]. However, there are gender differences in the severity of acts of violence and their consequences: men tend to commit the most severe acts and engage in sexual violence more often, and women engage in physical violence more often [35,36,37]. The literature identifies several risk factors for perpetrating and experiencing DV among emerging adults, including depression, low self-esteem, family dysfunction, substance abuse, childhood abuse, exposure to violence in the family, low socioeconomic status, and traditional gender roles beliefs [37,38,39]. Protective factors include interpersonal communication skills and emotional regulation skills [40].

### 1.3. The Role of Attitudes and Beliefs

Attitudes (characterized by overall assessments of an object or entity [41]) and beliefs (subjective judgments about what we consider true or false [42]) are distinct concepts, but they are not mutually exclusive [41,42]. A significant number of young people are involved in some form of violence in their dating relationships [6,7] and normalize or fail to recognize the various forms of violence, especially control, psychological violence, and violence through social media [43]. This legitimization may be associated with unrealistic beliefs about romantic love, namely the belief that there is a perfect partner, that love conquers all, and that jealousy is an expression of love [31]. These myths of romantic love, which are perpetuated by cultural narratives and the media, may stem from beliefs associated with gender roles, which still position women as submissive caregivers and men as primary providers [36,44]. When men hold beliefs about traditional masculine ideology, they may justify controlling, possessive, or aggressive behaviors as expressions of love, protection, or responses to women’s behaviors and provocations [35,45]. On the other hand, when women maintain a subordinate role, they may be more likely to give in to their partner’s demands, accept possessive and controlling behaviors, and remain silent in abusive relationships [35,45]. For example, there is currently a movement defending masculinity called The Red Pill, which spreads ideas such as that men should control their partner’s friendships, the clothes she wears, discourage her from attending university so she can devote herself to marriage and starting a family, and isolate them from influences that could jeopardize the relationship [36,44]. In a study that interviewed women whose partners held such beliefs, some participants reported “being constantly worried about their cell phone because their boyfriend would have them respond to all messages and calls immediately or he would end the relationship” [44] (p. 279) or even “when she did not respond to messages or calls on time, her partner accused her of infidelity and took her belongings and threw them out of the house” [44] (p. 281).

### 1.4. The Present Study

This qualitative study aimed to explore emerging adults’ beliefs and attitudes toward DV and to gather recommendations for prevention. To this end, we sought to answer three research questions (RQs):

**RQ1.**  *How do emerging adults define healthy intimate relationships?*
**RQ2.**  *How do emerging adults define and recognize dating violence?*
**RQ3.**  *What intervention strategies do emerging adults suggest, and what does this tell us about current approaches?*

## 2. Materials and Methods

### 2.1. Study Design

A qualitative study using focus groups was conducted to address the previously defined research questions, as this method allows for a reflective exploration of the phenomenon under study. In addition, interaction in a small group setting facilitates the sharing of ideas, reflection, and the emergence of new perspectives and contributions, with participants influencing each other’s views throughout the discussion [46].

### 2.2. Participants and Sampling

Two inclusion criteria were considered for participation in this study: (1) being between 18 and 25 years old and (2) speaking fluent Portuguese, regardless of nationality. Sixteen participants took part in this study, comprising 56% female (n = 9) and 44% male (n = 7), with an average age of 22.25 (*SD* = 1.73). Fifteen participants were Portuguese, and one participant was Chinese. In terms of religion, participants identified themselves as follows: catholics (38%, n = 6), agnostics (31%, n = 5), atheists (25%, n = 4), and 6% (n = 1) identified as “others.” Most participants (88%, n = 14) lived with their parents, 6% (n = 1) lived with other family members, and 6% (n = 1) lived independently. Geographically, and according to the Nomenclature of Territorial Units (NUTS), which corresponds to a territorial division system used by European Union member countries for statistical purposes, specifically NUTS II, 50% (n = 8) of the sample resided in northern mainland Portugal, 38% (n = 6) in the Autonomous Region of Madeira, 6% (n = 1) in the Lisbon metropolitan area, and 6% (n = 1) in Alentejo. All participants (100%, n = 16) reported that their place of residence was calm and safe. Regarding household income, 63% (n = 10) reported sufficient income, 31% (n = 5) reported comfortable income, and 6% (n = 1) reported insufficient income. All participants (100%) had completed compulsory education. In addition, the majority (56%, n = 9) of participants were full-time students, and 13% (n = 2) were working students. At the time of the study, all participants reported that they were not married and did not have children. A detailed characterization of the sample is provided in Table 1.

Participants were recruited through social media advertisements and using the non-probabilistic snowball method [47], whereby researchers made informal contact with their network of contacts via telephone calls or text messages. After expressing interest in participating in the study, each participant was asked to invite a member of the opposite sex to ensure gender equity. A Google Form was shared with participants via email, providing access to the digital informed consent form and the sociodemographic data sheet, which they were asked to complete before the focus groups.

### 2.3. Procedures and Data Collection

Three online focus groups were conducted by two members of the research team (ASC [Psychologist] and LF [psychology student]), via the Zoom platform (Zoom Video Communications, Inc., San Jose, CA, USA: https://www.zoom.com/pt (accessed on 24 February 2025)), each lasting approximately 60 min. The online format enabled us to bring together participants from different parts of the country. The first focus group served as a pilot (training) group to allow researchers to familiarize themselves with the script for conducting and managing the groups. Once this group met the participation requirements, it was included in the study sample. The groups consisted of 5 to 6 participants each, ensuring sufficient time for individual contributions during the discussions.

To obtain data, a semi-structured interview guide was developed (see Supplementary Table S1). This guide was based on empirical and theoretical literature and included seven open-ended questions that allowed exploration of three main themes: (1) healthy intimate relationships, (2) dating violence, and (3) prevention of dating violence. In addition to the interviews, a sociodemographic data questionnaire captured the demographic information of the participants, such as gender, age, nationality, marital status, educational level, professional status, residential area, household income, religion, relationship status, and the existence and number of children.

The sessions were recorded in audio and video, with free and informed participation via signed consent forms from all participants. The study was conducted in accordance with the ethical principles outlined in the Declaration of Helsinki [48].

### 2.4. Data Processing and Analysis

The focus group discussions were transcribed verbatim from the audio and video recordings. They were then carefully reviewed to gain a deeper understanding of their content. The complete data transcripts were imported into the qualitative analysis software MAXQDA (version 24).

Data analysis was performed using reflexive thematic analysis, a systematic method comprising six phases that enables patterns in qualitative data to be identified, organized, and described [49,50]. This approach is collaborative, interpretative, and reflexive, with the analytical process developed through ongoing discussions between researchers [49,50].

The thematic analysis was primarily conducted by one researcher (LF), and the interpretation of the data and the development of codes and themes were undertaken reflexively and collaboratively through regular intervision meetings with the entire research team, enhancing consistency and trustworthiness. In addition, two researchers (ACS and LF) independently analyzed selected excerpts from the transcripts and subsequently discussed their interpretations to develop a shared understanding.

In addition, the Consolidated Criteria for Reporting Qualitative Research checklist (COREQ) was used to ensure the research met standards for transparency and quality.

### 2.5. Ethical Considerations

All participants signed an informed consent form before participating in the study, which detailed the study objectives, procedures, data use, their right to withdraw, and confidentiality measures. Given the sensitive nature of the topic, participants were also provided with information on mental health support services and DV hotlines. The data were stored on the password-protected internal hard drive of the principal investigator’s laptop and deleted after analysis. All procedures followed the ethical principles of psychologists and the American Psychological Association (APA) code of conduct, and the Declaration of Helsinki, ensuring respect for participants’ autonomy, confidentiality, and voluntary participation [48]. The study was approved by the Scientific Committee of the Psychology Department of the University.

## 3. Results

**Figure 1:**
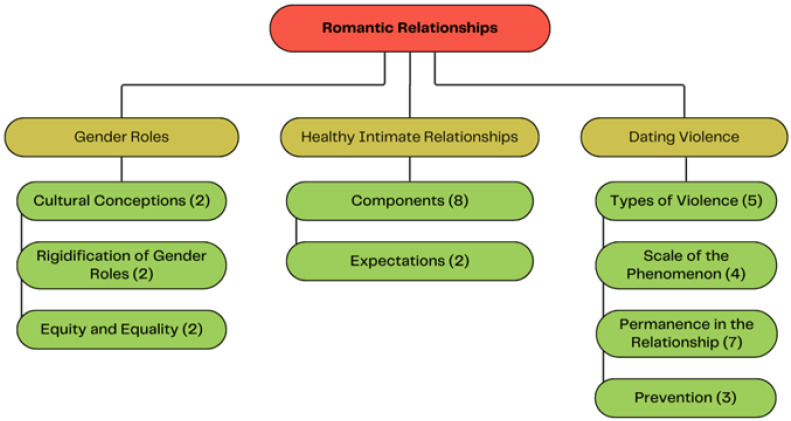
Thematic structure overview. Main theme (red), sub-themes (yellow), and codes and their variations (green).

Three main themes emerged from the thematic analysis (Figure 1): (1) gender roles, (2) healthy intimate relationships, and (3) dating violence, which are presented in detail throughout this section.

### 3.1. Theme 1: Gender Roles

Three sub-themes were developed from the Gender Roles (Table 2): (1) Cultural Conceptions, (2) Rigidification of Gender Roles, and (3) Equity and Equality.

#### 3.1.1. Cultural Conceptions

Participants discussed how cultural conceptions of gender roles shape romantic relationships, resulting in two codes: (i) Expectations and (ii) Education.

Cultural conceptions (norms/practices shared by a group) were found to affect *expectations*, particularly *gender-related expectations* and *gender-related expectations within the relationship*. These cultural norms shaped the values, ideas, and beliefs that subjects associate with the roles of men and women, both within and outside the relationship. Men were assigned the role of providing security and financial provision, being a stern figure, unlike women, who were assigned the role of caregiver and, consequently, an affectionate figure, as one participant illustrated:

Women stayed at home and took care of the children, and men were the breadwinners of the family. Even today, men are still seen as the ones who must provide security and who are the strength of the relationship, while women are seen as the caregivers, the more sensitive ones who wear their hearts on their sleeves. (S07, F).

These roles appear to be reinforced by education—described as intergenerational—which establishes different gender expectations, as another participant explained: “(…) men and boys are raised to be figures of power and to constantly suppress their emotions, while women are more fragile and therefore have reasons to show their fragility and express their emotions more easily.” (S08, F).

#### 3.1.2. Rigidification of Gender Roles

Two codes were developed based on the participants’ reports: (i) Wear and Tear and Saturation, and (ii) Emotional Unavailability.

The roles assigned exclusively to men or women would be detrimental to the relationship and culminate in *wear and tear and saturation*, which in turn would lead to *emotional unavailability*. For participants, the rigidification of gender roles is associated with emotional consequences that impact the couple’s well-being. One participant explained how role rigidity creates strain:

Even in domestic matters, it is important to have someone by our side to help us, so that everything does not fall on one person, because that ends up being a strain on only one of the two (…) when one person is worn out in the relationship, they can no longer give of themselves. (S09, F).

#### 3.1.3. Equity and Equality

Two codes were developed: (i) Sharing Tasks and Costs and (ii) Expectations Regarding the Partner.

Most participants share the idea that regardless of gender, the same rights, duties, and opportunities should be assigned fairly in romantic relationships. They demonstrated that the roles associated with men and women within the relationship are becoming blurred. Tasks previously performed solely by one gender can now be shared by both partners. Maintaining balance is key, which can be achieved through the *sharing of tasks and costs*. Participants provided concrete examples: “(…) today he pays, tomorrow I pay, or we split it 50-50.” (S10, F) and “(…) at dinner, one can make the other clean up, or both can be involved in it.” (S15, M).

In addition, the results revealed that expectations regarding the partner have become more egalitarian, as subject 10 stated: “I am not the kind of woman who expects the man to do everything.” (S10, F).

### 3.2. Theme 2: Healthy Intimate Relationship

Participants articulated consistent expectations for healthy intimate relationships. Two sub-themes emerged from the data (Table 3): (1) Components and (2) Expectations.

#### 3.2.1. Components

Eight codes were developed in relation to the components of healthy intimate relationships, with *respect*, *trust*, and *individuality* most frequently cited. Participants highlighted the importance of mutual respect and trust as essential for maintaining a healthy intimate relationship. They indicated that it is important to have someone by their side who respects their feelings, opinions, and boundaries-someone they can trust and who trusts them-as exemplified by the following quote: “I would say that the basis is mutual respect, and I think trust too, honesty, I think those are the pillars of a healthy relationship.” (S16, F). In addition, they highlighted the importance of space within the relationship for each partner to maintain their individuality: “For me, we must have a life outside our relationship.” (S03, F). Less frequently mentioned were *freedom and independence*, *empathy*, *sincerity*, *honesty*, and *loyalty*. Although less frequently mentioned, participants emphasized the relevance of transparency in a relationship, expecting it to be guided by values such as honesty, sincerity, and loyalty, which should be maintained permanently, as illustrated by the following quote: “I think that above all, even knowing that sometimes it may not be a good subject, it may be a bad motive, I would prefer them to be sincere with me.” (S12, F).

#### 3.2.2. Expectations

Two codes were developed in relation to Expectations: (i) Emotional Support and (ii) Couple Functioning/Dynamics.

*Emotional support* includes a set of codes, such as expectations that the chosen person will be a source of *understanding*, *support/care,* and *complicity*. The subjects in the sample expect their partner to be a presence in their lives and that their presence will bring well-being, be associated with positive interactions such as support/care, understanding, and complicity, and others that are presented below. These expectations are clearly expressed:

Whether something good or bad happens, I only think about telling that person, because that person will understand me, that person will help me, and that person will be there for me if things go well or if things go badly. (S10, F).

In addition, they expect *friendship*, as expressed in the following illustration: “(…) I think that first and foremost, if we are friends with each other, that is already a starting point for a healthy relationship.” (S09, F), and *comfort*, *listening*, *kindness*, *acceptance*/*appreciation*. Participants want their partner to accept and value them as a person and contribute to their personal growth. Finally, some participants mentioned expecting their partner to support them in *raising their self-esteem*, as illustrated by the following quote: “Always raising each other’s self-esteem has to be a balance. We are not always well, but the other person also has to know how to raise their partner’s self-esteem.” (S10, F). Comfort, understanding, and acceptance/appreciation are interrelated. When partners feel understood, accepted, and appreciated in their sharing, this fosters a sense of comfort and encourages further sharing. The importance of being understood without judgment is illustrated in the following excerpt:

I was thinking about the fact that in a relationship, I would expect to be able to share moments of fragility, more intimate moments, and everything else, and to be able to do so without feeling uncomfortable or feeling that I am doing something wrong, and that the other person accepts it. (S14, M).

Regarding *couple functioning/dynamics*, the most prominent codes are *communication/problem solving*, *boundaries*, and *knowing one’s partner*. Communication is considered essential for maintaining a healthy intimate relationship, as it enables problems to be resolved:

The important thing is to be able to talk, to say that it happened, that you were hurt, and to make sure that both of you understand that it can’t happen again, that you have good communication so that you understand where you went wrong. (S13, F).

In addition, it also establishes the boundaries of a relationship, as seen in the following quote:

It is important to set boundaries when starting a relationship. That’s it, we always have to have that conversation, and with a lot of communication, we end up defining each other’s boundaries (…) by talking, we can define and find a balance between what one wants and what the other wants and what we both don’t want. (S09, F).

Getting to *know one’s partner and sharing ideas and preferences* were considered relevant by participants and contribute to the adaptation partners must make to each other: “I think that a relationship depends a lot on our partner knowing us and knowing how to respond in the best way to us.” (S15, M). Getting to know your partner or letting your partner get to know you would be related to authenticity and spontaneity, as highlighted by subject 08: “(…) with our partner, I think that to have a healthy relationship, it is important to be willing to show everything good and less positive about ourselves, not to be afraid of being vulnerable.” (S08, F). In a healthy intimate relationship, participants expect *complementarity* and *reciprocity*. When faced with moments of greater vulnerability, participants expect their partners to complement them, as evidenced by the quote: “(…) I moments when one person is feeling down, and the other will have to give a little more.” (S05, M). Finally, they expect reciprocity in interactions and actions—that is, a return for the gestures made by their romantic partner: “(…) be there for me as I know I will be there for her in every way.” (S10, F).

### 3.3. Theme 3: Dating Violence

This theme captures participants’ awareness, recognition, and minimization of DV, alongside their suggestions for prevention. Four sub-themes are developed (Table 4): (1) Types of Violence, (2) Extent of the Phenomenon, (3) Permanence in the Relationship, and (4) Prevention.

#### 3.3.1. Types of Violence

From the information provided by participants regarding types of violence, five codes were developed: (i) Physical violence, (ii) Sexual violence, (iii) Cyber violence, (iv) Psychological violence, and (v) Relationship dissatisfaction.

Within the physical violence code, two variations related to aggressive behaviors were identified: *slapping* and *pushing*. Within the *sexual violence code*, two variations described acts such as *forcing a kiss* and *forcing sexual intercourse*. Regarding cyber violence-a form of violence that occurs online-obtaining a password was considered an act of control that leads to loss of privacy, as subject 01 noted: “(…) giving the password to the other partner, I think that might be a bit too much, I think everyone should have their privacy.” (S01, M).

Within *psychological violence*, participants highlighted verbal violence (*accusations* [13%; n = 2], *insults* [13%; n = 2], and *criticism and derogatory comments* [50%; n = 8]) and controlling behaviors (monitoring, manipulation, love bombing, and restrictions on freedom and individuality), described as particularly harmful to autonomy and self-esteem. The accusations were linked to suspicion and lack of trust, while the insults and derogatory comments were related to personal value and self-esteem, as can be seen in the statement by subject 10:

We get ready, and the person makes a super negative comment, and for them it’s a joke, they’re laughing, it was just a comment, ‘I was teasing you,’ and we swallow it, swallow it, because it hurts us, because my self-esteem goes up the stairs and down the elevator. (S10, F).

Reports of *disapproval* were also verified: “(…) it all starts with a look of disapproval, and the person feels compelled to do something to gain their partner’s approval.” (S15, M); *devaluation*: “(…) we are nothing if we don’t have them.” (S10, F); and *decreased self-esteem*: “(…) it goes through everything that can diminish the other person’s self-esteem or self-concept.” (S07, F).

*Controlling behaviors* were also noted in participants’ reports, such as monitoring location, activities, choice of clothing, and friends, which participants associated with a *loss of freedom and individuality*. As the participants illustrate, this involves: “(…) controlling in any way what the other person does, with whom they do it, how they do it.” (S15, M) or: I didn’t think I was controlling my boyfriend, I thought it was normal, but I think it’s not because of the conversation we had (…) he says he’s going to dinner with friends, he even sent me a photo because I’ve manipulated him a bit. (S11, F).

*Manipulation* and *love bombing* were also described by participants as strategies used by one partner to control and hurt the other, as exemplified by subject 13: “I will give you my love in excess so that you like me and then I will hurt you.” (S13, F). *Inducing insecurity*, although less frequent, was also reported by participants: “putting my partner in an unsafe home, making her feel that the relationship was not safe for her.” (S04, M). Finally, one participant identified *relational dissatisfaction* as a type of violence, associating the frustration of needs with violence in dating, as illustrated by subject 07:

From the moment you look at the person next to you and realize that you don’t see yourself in them and that they are not respecting you, not meeting your minimum needs, not ensuring your satisfaction, and that it would be better to be alone. (S07, F).

#### 3.3.2. Extent of the Phenomenon

From the information provided by participants regarding the extent of the phenomenon, four codes were developed: (i) Very Frequent, (ii) Frequency/Consequences of gender-based victimization, (iii) Resources/support, and (iv) Difficulty vs. Ease in identifying types of violence.

Participants considered DV to be a *very frequent phenomenon*, highlighting that it is often *romanticized* in movies, books, and the *judicial system*, as exemplified by one participant: “(…) society, like movies and books, romanticizes these actions, but such behaviors should not be romanticized.” (S11, F).

According to the participants, changes in the law have contributed to an increase in the number of reported cases, which consequently increases the prevalence of DV, as explained by subject 08: “(…) it is not that the cases themselves are increasing, but rather that we can report these cases, which makes them more frequent.” (S08, F). At the same time, they also emphasized the *devaluation and unconsciousness* of early warning signs in potentially abusive relationships:

The people themselves who are in this type of relationship devalue it, or when they see this type of behavior in their partner, they devalue it, because they think it will always happen to someone else, it will never happen to me, they will never hit me, it will never get to that point for me, and they let things slide until it becomes a violent relationship. (S07, F). Many participants also noted a lack of awareness among those in relationships about the violence they are experiencing: “the people themselves are unaware that they are in this vicious circle.” (S03, F). Regarding the *frequency/consequences of gender-based victimization*, participants believed that women are victimized more often and that the consequences of violence perpetrated by men tend to be more severe, due to the greater physical strength they attribute to men. These thoughts are clearly identified in the following illustration: “(…) I believe that, by nature, there may be more cases of women suffering because, naturally, men tend to be stronger.” (S09, F). They also reported that the *resources/support* available to victims are more focused on women, and were unable to identify equivalent responses for males: “(…) I am sure there are shelters for women who suffer domestic violence and so on, but I don’t know of anything that supports men.” (S09, F).

Finally, concerning the *difficulty* vs. *ease of identifying types of violence*, physical violence was considered the easiest to identify compared to other forms, particularly psychological violence, which participants described as more subtle and difficult to identify. We can verify this information in the following reports: “(…) with physical violence, it’s something that we look at and see that it didn’t work out or that it’s a problem between the couple, that things are really bad.” (S05, M), and “it starts with psychological violence, which is more complicated to detect whether someone suffers from it or not.” (S06, M). Although participants recognized various types of violence, they assumed that when they think of dating violence, physical violence is the first image that comes to mind, justifying that this attribution reflects what has been conveyed to them: “what we are taught is that DV is physical (…) a misconception that is conveyed to us.” (S10, F).

#### 3.3.3. Permanence in the Relationship

From the information provided by participants regarding their decision to remain in the relationship, seven codes were developed: (i) Emotional dependency, (ii) Financial dependency, (iii) Couple dynamics, (iv) Expectation of change, (v) Removal of blame, (vi) Normalization, and (vii) Low self-esteem.

*Emotional* and *financial dependency* were mentioned as reasons for remaining in abusive relationships, especially when children are involved. Participants also reported that, in some couples, violence becomes part of the relationship dynamic and may not be perceived as a problem by those involved, as exemplified by the following quote:

I find myself in a situation like this and only then am I able to reflect that ‘wow, when I saw this in others it was ridiculous, but when I went through it myself, it was acceptable or forgivable, or there was an excuse for it’. (S03, F).

The reasons most frequently mentioned by participants were the *removal of blame* and *normalization* of acts of violence by victims who believe in nurturing feelings between the couple and the *expectation of change* for love, as expressed in the following quote: “This will change, this will stop, with me it will be different.” (S10, F). *Low self-esteem* was also cited as one of the reasons, as stated in the quote: “The person themselves does not value themselves enough to realize that they deserve more than that and that they can have more than that.” (S07, F).

#### 3.3.4. Prevention

Three codes related to prevention were developed: (i) Intervention strategies, (ii) Preventive tools, and (iii) Strategy change.

In the *intervention and prevention strategies*, various methods emerged, such as the *investigation*, which, according to subject 09, prevention would involve studying beliefs to shape them:

Given the current emphasis on mental health, studying children could be an effective way to shape some of their thoughts or beliefs that they consider natural, considering what they experience at home. (S09, F).

*Psychological intervention* was also mentioned by participants, who emphasized the importance of seeking help from specialists. More frequently, *familiarization with the topic* was cited. Participants agreed that it is necessary to address the issue and inform the population to raise societal awareness and educate people about DV, thereby preventing its occurrence. In the same vein, they considered it pertinent for awareness-raising actions to address the issue by explaining what love is, as illustrated by subject 16:

Often, people don’t know what the definition of love is, they don’t know what healthy love is because they’ve never seen it (…). So, I would say that the best way to implement it is to talk more and discuss what it means to love yourself and other people. (S16, F).

In addition, participants found the use of a mobile application to prevent DV interesting. Firstly, they indicated that it would be relevant to include an explanation of DV by *themes and dimensions*, such as the characteristics of an abusive relationship, sharing strategies for overcoming it: “I think essential things that should be included are factors or actions that lead one to think that one is in a toxic relationship.” (S16, F); “it could also be interesting to share strategies, that is, what the person did to get out of this relationship.” (S08, F); *support contacts*, whether psychologists, protection and security services, associations, or lawyers; *helpline* similar to those that exist for other issues, available 24 h a day; *testimonials* that would make it easier for potential victims to identify warning signs or recognize violent behavior, and contribute to a feeling of acceptance, as exemplified by subject 07:

To draw attention to the issue, make help available in advance, and share personal testimonies or contributions ahead of time, because sometimes just hearing that we are not alone in going through this, and that the situation may be problematic, is enough to awaken awareness within us. (S07, F).

Participants considered *open chat* an advantageous communication tool, as it enables victims to exchange experiences in real time, fostering a sense of belonging and solidarity: “They can share bits of their problems and basically create a community, so they feel more included and more open to talking about their problems.” (S14, M). They also suggested the use of *games*, incorporating explanations of DV, warning signs, examples of violent behavior, coping strategies, and testimonials, as subject 07 noted: “It’s like a quiz where you answer questions about what you have in your relationship, what you don’t have in your relationship, and then you get the final answer.” (S09, F).

Participants also considered that DV prevention could occur through *partnerships with brands and influencers*, *social networks*, since these channels can reach a wider audience, as illustrated by the following quote: “Partnerships with influencers, with people who have a larger audience, would be a good way to build this audience (…) social media is the easiest formula.” (S03, F). The *support network* was considered essential for DV prevention, as people close to victims can help them recognize warning signs or end the relationship. Participants viewed formal education as essential, with 19% believing the topic should be discussed in schools, which they saw as institutions playing a fundamental role in educating students and addressing issues impacting young people:

There should be a subject in the curriculum for young people that makes it compulsory to talk about these issues, to debate these issues and to encourage people to think critically (…) I think that education has several important areas, but I think that developing humans, developing people who are aware of this type of behavior, both for ourselves and for others (…) makes a lot of sense that this is something that should be included in the curriculum, since if you have to learn to do math, you should also learn how to interact with people and communicate in a non-violent way and in a way that expresses what you really want. (S07, F).

Beyond school, participants believe that the topic should be addressed in other circles, such as *discussion groups* organized by different bodies: “A gathering of young people in a circle, simply talking about the topic, having some questions to address, discussing this topic, and getting them to talk to each other, to share experiences” (S07, F).

Finally, some participants suggest a *change in strategies*, mainly in the work carried out with young people in schools, which participants consider inadequate, an intervention that does not take into account the age group of the target audience and is unable to capture the interest and attention of young people, as illustrated by the following quote:

We have these actions for very young people, and I remember seeing everyone around me always uninterested, laughing, the teachers don’t pay attention, I think this type of lecture should be done, but with fewer people, going to classrooms, with fewer people, but more often, so that it would be easier to capture the attention of the people who are listening. (S10, F).

Participants also called for greater awareness among parents and society at large, noting that all systems influencing young people contribute to shaping beliefs about DV: “(…) work with parents should also be emphasized.” (S02, M); “(…) there must not only be this approach to children through school, but there must also be this approach to people in general.” (S13, F).

## 4. Discussion

The present study aimed to understand emerging adults’ attitudes and beliefs regarding DV and provide recommendations on its prevention. Thus, the results of this study reveal a duality in the gender roles representations, where the persistence of gender stereotypes coexists with a growing appreciation of equity and equality, reflecting Portuguese data showing that young people simultaneously hold attitudes that support gender equality and traditional beliefs about the roles of men and women. These beliefs have been associated with the normalization of DV [51]. The literature also shows that contexts of greater gender inequality have higher prevalences of intimate partner violence and that men tend to internalize gender role stereotypes more, while women are more in favor of an equal division of household chores [52,53]. However, our results show that both female and male participants adopted attitudes favorable to gender equity, in line with the study by Sonkaya and Öcal [54] conducted with 1082 university students, which similarly found participants adopting progressive and egalitarian attitudes toward gender roles, suggesting a shift in cultural conceptions.

The participants’ responses highlighted mostly positive expectations of healthy intimate relationships, describing them as based on respect, trust, emotional support, and communication. This supports the existing literature suggesting that trust, respect, open communication, and emotional connection are essential characteristics of healthy romantic relationships [55]. Additionally, our results show that participants considered it essential to know their romantic partner intimately and share ideals to establish a healthy intimate relationship. Similar attitudes toward gender roles (such as the division of housework and childcare) are fundamental to everyday issues. A study of young couples together for up to five years without children found that sharing attitudes toward gender roles leads to greater stability and satisfaction, while divergence generates conflict, lower satisfaction, and higher separation risk [53]. In addition, the literature shows that these characteristics can be protective for the relationship, since gender inequality correlates with violence prevalence [52] and its normalization [56]. Our results also showed that participants value the preservation of their identity and individuality, reporting that a healthy intimate relationship is a space for mutual support, understanding, complicity, and where they can be themselves. These findings align with some studies. For example, a study conducted with emerging adults showed that healthy romantic relationships serve as a context for exploring and consolidating identity, as they promote self-confidence, dependence, and autonomy [57]. Moreover, the literature also reinforces that communication is essential for problem-solving and for setting boundaries [55,57,58], which reinforces the importance of interpersonal skills in building healthy relationships and resolving conflict.

Concerning dating violence, the participants demonstrated awareness of various forms of violence, believing that, due to its physical nature, women suffer more violence in intimate relationships and that violence perpetrated by men is more severe, as confirmed by Caridade and Machado [35]. The participants also recognized male victims and the lack of support resources for them, consistent with literature showing that both genders can be victims and perpetrators, but men seek help less often due to shame, fear of ridicule, and inadequate support services [59]. Participants were also aware of the seriousness of acts of violence, which, according to Martin-Fernández et al. [60], is associated with less acceptability and victim blaming, and greater willingness to intervene. However, this knowledge did not prevent them from remaining in intimate relationships marked by violence. The literature identifies financial dependence, investment in the relationship, fear of leaving the partner, love, and hope for change as reasons for remaining in abusive relationships, which help explain why, despite their awareness, some participants normalized or minimized, especially psychological forms of violence in their own relationships [45]. Our results revealed several romantic love myths (omnipotence, the perfect partner, need for a couple, eternal passion, exclusivity, marriage, jealousy, and ambivalence) that are associated with violence in intimate relationships, because they prevent recognition of certain actions as violent [61]. According to Grané-Morcillo et al. [62], these myths are highly prevalent among young people, particularly omnipotence, soulmates, exclusivity, and self-sacrifice. In this study’s population, the myth of the perfect partner was most prevalent, with the partner viewed as the person who will satisfy all needs [62]. Although positive expectations could lead to healthy functioning, they can also lead to disappointment and marital dysfunction [63]. The expectation that one’s partner can satisfy all the needs previously satisfied by a wider group of people can lead to feelings of dissatisfaction in the relationship, explaining the increase in conflict within the couple [64]. Feelings of love and expectations of change can contribute to justifying acts of violence. This belief can be reinforced by describing perpetrators as being under the influence of uncontrollable emotions, legitimizing the myth of ambivalence [65].

About preventive strategies, participants mentioned school interventions on DV as a form of prevention. They highlighted the need to change formal education strategies, developing work closer to the student community, with greater frequency and smaller groups. Specifically, they suggested creating a course where it would be mandatory to talk regularly about interpersonal relationships. Literature also found a need to change interventions by introducing behavioral changes through the development of skills and competencies [66]. According to De la Rue et al. [66], one-off and isolated interventions increase knowledge, helping them recognize abusive behaviors and discover available resources. However, of the 23 studies analyzed, three showed no change in levels of violence perpetration, and five that assessed victimization showed little effect, explaining the discrepancy between high awareness and perpetration behaviors [66].

Regarding the prevention approaches of DV, the participants clearly demonstrated that DV must be adapted to their reality and interests. In this sense, they made some suggestions for prevention. They recommended the creation of a school discipline in which it would be mandatory to talk regularly and systematically about topics such as DV and interpersonal relationships. This suggestion aligns with the literature, which recognizes the school context as a privileged environment for primary prevention [67].

Finally, participants highlight the role of digital media and technologies in preventing DV. This perspective is consistent with the literature, which shows that emerging adults spend a lot of time online, making digital approaches more appealing and accessible than traditional approaches [7,68]. In this regard, participants suggested two strategies in particular: (i) creating campaigns on social media or with influencers, and (ii) developing a mobile application to address the issue in a fun and interactive way. Despite the enormous reach offered by social media, evidence regarding its use in DV prevention campaigns remains unclear, with a lack of rigorous evaluations of its effectiveness [69,70]. The fact that participants reported that a mobile application would allow them to learn about the topic confidentially, independently, without fear of judgment, and without shame reflects the advantages attributed to digital interventions as tools to be used to address sensitive topics related to mental health and DV [71,72]. By emphasizing the need to adapt prevention strategies to their interests, participants reinforce the suggestions of several studies, which highlight the importance of participatory and user-centered approaches, such as co-constructing prevention programs with the recipients themselves, ensuring that they are engaging, attractive, and relevant to the target audience [72,73]. Similarly, digital technologies, particularly mobile applications and games, show growing potential as tools for behavioral change and social impact [68,74,75]. In addition, they have also been shown to be effective in raising awareness of DV and reducing the social norms and beliefs that support DV [76,77,78,79]. A study analyzing emerging adults’ perspectives on the use of game-based mobile applications for DV prevention suggests that interactive digital approaches can promote deeper engagement, encourage empathy and self-reflection, and offer a more flexible and accessible solution [72]. Thus, our findings suggest that technologies may play an important role in preventing DV, specifically among emerging adults.

### 4.1. Implications for Preventive Practice

The results of the present study have relevant implications for prevention and practice, insofar as emerging adults expressed the need for interventions tailored to their developmental characteristics, and valued the use of digital tools in the prevention of dating violence. Prevention programs implemented in school settings showed an impact on increasing knowledge about the phenomenon [80]. However, they did not demonstrate statistically significant effects on attitudes and behaviors [80]. This discrepancy between knowledge acquisition and attitudinal/behavioral change suggests limitations in traditional awareness-raising and prevention approaches. Factors such as interaction, immersion, and motivation, which are often associated with experiences provided by mobile applications or games, tend to be absent from conventional interventions [81]. A study aimed at developing an engaging game as an alternative to existing intervention programs highlighted the relevance of using tools that are integrated into the daily lives of emerging adults, a population particularly affected by dating violence. Following participation in the game, 89% of the participants who completed the semi-structured interview reported the experience as gratifying, considering it superior to traditional intervention programs. Additionally, participants reported feeling more engaged and challenged, emphasizing the promotion of critical thinking during decision-making situations [81]. Similarly, mobile prevention applications have shown potential as a promising strategy in combating dating violence [82]. Several proposals put forward by participants, namely the development of digital resources providing information on the definition of dating violence, its different types, methods of identification, and the characteristics of healthy relationships, were operationalized and evaluated in a study with 354 university students. The findings indicated a decrease in the acceptance of violence and an increase in knowledge about the phenomenon, although the results were not statistically significant, reinforcing the need to further refine these approaches [82]. Currently, existing prevention applications are primarily designed for individuals in crisis situations, relying on safety planning and revictimization prevention strategies [83]. These tools are characterized by the provision of privacy and anonymity and, for this reason, constitute a preferred means for adolescents and emerging adults to discreetly access integrated educational resources [83].

Overall, findings from international studies point to a reduction in risk associated with the use of digital resources, which justifies particular attention to the results of the present study to support the adoption and adaptation of similar measures in the national context, with a specific emphasis on the development of dynamic tools, such as games and preventive applications, in the fight against dating violence.

### 4.2. Limitations

This study has several limitations that should be considered when interpreting the findings. The research method involved focus groups, which are advantageous because they enable reflective exploration of participants’ beliefs and attitudes, and the sharing of ideas and perspectives within a small group. However, discussing responses in a group setting may have inhibited participants from fully exploring their attitudes and beliefs or encouraged socially desirable responses.

Three focus groups were conducted, and the results across groups were highly cohesive, suggesting theoretical saturation. On the other hand, this cohesion may stem from sample similarities; although heterogeneous in terms of gender, the sample was relatively homogeneous in other characteristics. Most participants had a high level of education, were enrolled in academic courses, and were financially dependent on their parents. Furthermore, none were married or had children. This sample characterization aligns with the sociodemographic profile of emerging adults in the literature, which is a strength of the study. One limitation was the absence of control, for ethical reasons, over participants’ current or previous involvement in abusive relationships.

The high homogeneity in the sample, particularly regarding the participants’ high educational attainment, may limit the transferability of the findings to populations with more diverse educational backgrounds. Therefore, the results should be interpreted with caution, as participants in this study may exhibit higher levels of awareness than those in more heterogeneous populations. Future research should include samples with greater educational diversity to enhance the transferability of the findings.

The interview script used broad questions that encouraged reflection and idea exchange among participants. The online format of the focus groups proved advantageous, as it enabled participants from insular Portugal (e.g., Madeira) to take part, reducing constraints related to time and travel. However, the participants’ geographical origins lacked diversity, limiting the generalizability of results to the broader population.

Finally, although reflexive procedures were employed throughout the data analysis process, and two researchers independently coded excerpts from the transcripts, the fact that the analysis was primarily conducted by a single researcher may have influenced the interpretation of the data.

## 5. Conclusions

This study aimed to understand attitudes and beliefs toward DV among emerging adults and revealed that both male and female participants advocate for equality and equity in gender roles, signaling a shift in cultural conceptions.

Although participants demonstrated a high awareness of what DV is, the different types of violence, and the severity of the phenomenon, it was found, through the sharing of experiences, that these factors were not sufficient to prevent them from becoming involved and remaining in violent intimate relationships. Participants highlighted unawareness, normalization, and excuse-making as reasons for remaining in violent relationships, which, according to the results of the discussion groups, were associated with the myths of romantic love and the idealization of relationships. This idealization is verifiable when expectations for a relationship are solely positive, with romantic relationships being associated with well-being and the absence of conflict, which is inevitable in human relationships.

This study has helped to fill the gap in the literature on the attitudes and beliefs of emerging adults towards DV and will have practical implications for the prevention of DV. The ability demonstrated by emerging adults to reflect on the subject and the contributions they have made should alert stakeholders to the need to introduce changes in the field of DV prevention, such as changes in school-level interventions and the participation of different entities and bodies with legislative power.

Understanding attitudes and beliefs is crucial for examining DV from emerging adults’ perspectives, a developmental stage marked by increased DV prevalence. Yet, even among those with beliefs and attitudes less favorable to DV, such attitudes alone do not prevent them from becoming involved in abusive relationships. Thus, intervention strategies must be rethought, alongside the transmission of ideologies about dating that hinder violence recognition and encourage remaining in abusive relationships.

## Figures and Tables

**Table 1:** Sample characterization of the sample.

Code	Gender	Age	Professional Situation	Level of Education	Course/Profession	Residential Area	Relational Situation	Relationship Duration
S01	M *	19	Student	S *	Bachelor’s in Psychology	Semi-urban	Dated	3 months
S02	M	19	Student	S	Bachelor’s in Psychology	Semi-urban	Dated	1 year 3 months
S03	F *	21	Unemployed	B *	Graphic Design	Semi-urban	Dated	1 year
S04	M	21	Worker	S	Ambulance Crew Member	Rural	Dating	1 year 5 months
S05	M	22	Student	B	Master’s in Computer Engineering	Semi-urban	Never dated	-
S06	M	22	Student	B	Master’s in Computer Engineering	Urban	Dating	6 years
S07	F	22	Student	B	Master’s in Psychology	Urban	Dating	2 years
S08	F	22	Student	B	Master’s in Psychology	Rural	Dating	6 years
S09	F	22	Working Student	S	Bachelor’s in Radiology	Semi-urban	Dating	7 months
S10	F	23	Worker	S	Tourist Guide	Rural	Dated	5 months
S11	F	23	Student	B	Master’s in Industrial Engineering	Urban	Dating	1 year 10 months
S12	F	23	Student	S	Physical therapy	Rural	Never dated	-
S13	F	23	Worker	B	Graphic Design	Semi-urban	Dating	3 months
S14	M	24	Unemployed	M *	Computer Engineering	Urban	Never dated	-
S15	M	25	Student	B	Master’s in Computer Engineering	Urban	Dating	10 years
S16	F	25	Working Student	M	Design	Urban	Dated	1 year

* M = Male; F = Female; S = Secondary education; B = Bachelor’s degree; M = Master’s Degree.

**Table 2:** Frequency of codes—Theme 1: Gender roles.

Sub-Themes	Codes and Variations	n	R
Cultural Conceptions	Expectations		
	Gender-related	2	3
	Gender-related issues within the relationship	4	4
	Education	3	3
Rigidification of Gender Roles	Wear and Tear and Saturation	3	3
	Emotional Unavailability	1	1
Equity and Equality	Sharing Tasks and Costs	6	10
	Expectations Regarding the Partner	4	6

**Table 3:** Frequency of codes—Theme 2: Healthy Intimate Relationship.

Sub-Themes	Codes and Variations	n	R
Components	Respect	7	9
	Honesty	2	2
	Sincerity	1	2
	Loyalty	1	1
	Trust	6	7
	Empathy	2	2
	Freedom and Independence	2	3
	Individuality	5	5
Expectations	Emotional Support		
	Understanding	6	10
	Support/Care	4	6
	Complicity	4	4
	Friendship	3	4
	Comfort	2	4
	Listening	3	3
	Kindness	3	3
	Acceptance/Appreciation	2	2
	Raising their self-esteem	2	2
	Couple Functioning/Dynamics		
	Communication/Problem-solving	7	16
	Boundaries	6	10
	Adaptation	3	3
	Know one’s partner	4	5
	Sharing ideas and preferences	2	2
	Complementarity	2	3
	Reciprocity	3	3

**Table 4:** Frequency of codes—Theme 3: Dating Violence.

Sub-Themes	Codes and Variations	n	R
Types of Violence	Physical violence		
	Slapping	1	3
	Pushing	1	3
	Sexual violence		
	Forcing to kiss	1	1
	Forcing to have sexual intercourse	1	1
	Cyberviolence		
	Loss of privacy	1	1
	Psychological violence		
	Verbal violence		
	Accusations	2	2
	Insults	2	2
	Criticism and derogatory comments	8	9
	Control		
	Inducing insecurity	1	1
	Love Bombing	2	2
	Manipulation	3	3
	Loss of Freedom and Individuality	8	14
	Disapproval	2	2
	Devaluation	4	5
	Decreased self-esteem	3	3
	Relationship dissatisfaction	1	2
Extent of the Phenomenon	Very frequent		
	Unconsciousness	9	13
	Devaluation	1	1
	Romanticization of dating violence	1	1
	Judicial system	3	5
	Frequency/Consequences of gender-based victimization	3	4
	Resources/Support	1	2
	Difficulty vs. Ease in identifying types of violence	6	8
Permanence in the Relationship	Emotional dependency	2	4
	Financial dependency	1	3
	Couple dynamics	2	4
	Expectations of change	3	4
	Removal of blame	3	7
	Normalization	5	10
	Low self-esteem	1	1
Prevention	Intervention strategies		
	Digital resources		
	Themes and dimensions	5	5
	Playful material	2	2
	Helplines	2	2
	Support contacts	7	9
	Open chat	5	5
	Testimonials	5	6
	Investigation	1	2
	Psychological intervention	3	6
	Familiarization with the topic	9	11
	Preventive tools		
	Partnerships with brands and influencers	2	3
	Social networks	1	2
	Laws	1	1
	Support network	3	3
	Formal education	3	4
	Discussion groups	1	2
	Change in strategies	5	6

## Data Availability

The data that support the findings of this study are available from the corresponding author upon reasonable request.

## Supplementary Materials

The following supporting information can be downloaded at https://www.mdpi.com/article/10.3390/ijerph23030294/s1. Table S1: Semi-structured interview guide.
