# Supplementary material for: Meeting Prevention Beyond Awareness: A Qualitative Study Exploring Attitudes and Beliefs Towards Dating Violence and Prevention Among Emerging Adults
Source: Int J Environ Res Public Health. 2026 Feb 27;23(3):294. doi: 10.3390/ijerph23030294 (PMC13026653; doi:10.3390/ijerph23030294)
Supplement: Supplementary file 1 [file ijerph-23-00294-s001.zip › ijerph-4157760-supplementary.pdf]

Supplementary Table S1. Semi-structured interview guide.

Phase	Guiding question
Group introduction	Presentation of the study and objectives<br>Explanation of confidentiality, anonymity, and consent<br>Brief introduction of participants
Discussion	1. How would you describe a healthy relationship?<br>2. Do you think there are differences in how men and women are expected to behave in a relationship?<br>3. When we talk about dating violence, what things come to mind?<br>4. Do you think dating violence is common or rare? Why?<br>5. Are there situations or behaviors within a dating relationship that are not considered a form of violence? Can you give examples and explain?<br>6. What do you think could be done to prevent violence in dating relationships?<br>7. What approaches or tools do you think could be used to prevent dating violence?
